# Enhancing the chemical transformation of *Candida parapsilosis*

**DOI:** 10.1080/21505594.2021.1893008

**Published:** 2021-03-17

**Authors:** Tibor Németh, Joshua D. Nosanchuk, Csaba Vagvolgyi, Attila Gacser

**Affiliations:** a Department of Microbiology, Faculty of Science and Informatics, University of Szeged, Szeged, Hungary; b Division of Infectious Diseases, Department of Medicine, Albert Einstein College of Medicine, Bronx, New York, USA; c Department of Microbiology and Immunology, Albert Einstein College of Medicine, Bronx, New York, USA; d MTA-SZTE Lendület Mycobiome Research Group, University of Szeged, Szeged, Hungary

**Keywords:** *Candida parapsilosis*, chemical, transformation, optimization, freezing, depositing, protocol

## Abstract

*Candida parapsilosis* is a leading cause of invasive mycoses and the major cause of nosocomial fungaemia amongst low and very low birth weight neonates. However, the molecular and physiological characteristics of this fungus remain understudied. To advance our knowledge about the pathobiology of this pathogen, we sought to develop and validate an effective method for chemical transformation of *C. parapsilosis*. Chemical transformation is the primary procedure for introducing foreign DNA into *Candida* yeast as it requires no special equipment, although its performance efficacy drops rapidly when the size of the transforming DNA increases. To define optimal conditions for chemical transformation in *C. parapsilosis*, we selected a leucine auxotroph laboratory strain. We identified optimal cell density for transformation, incubation times, inclusion of specific enhancing chemicals, and size and amounts of DNA fragments that resulted in maximized transformation efficiency. We determined that the inclusion of dimethyl sulfoxide was beneficial, but dithiothreitol pretreatment reduced colony recovery. As a result, the modified protocol led to a 20–55-fold increase in transformation efficiency, depending on the size of the transforming fragment. We validated the modified methodology with prototrophic isolates and demonstrated that the new approach resulted in the recovery of significantly more transformants in 5 of 6 isolates. Additionally, we identified a medium in which transformation competent yeast cells could safely be maintained at −80°C for up to 6 weeks that reduces laboratory work and shortens the overall procedure. These modifications will significantly aid further investigations into the genetic basis for virulence in *C. parapsilosis.*

## Introduction

Fungal infections represent a global medical threat with nearly one billion cases occurring annually worldwide. Out of these, approximately 150 million infections represent serious conditions and 1.6 million are fatal. *Candida* species are responsible for the vast majority of hospital acquired systemic fungal infections with *C. albicans* being the most frequently encountered species followed by *C. glabrata, C. parapsilosis, C. tropicalis* and *C. krusei* [1]. Over millennia, these species have gained specific properties to persist in/on the host and cause a disease, particularly in the setting of a compromised host immune system. Amongst these species, *C. parapsilosis* tends to infect primarily low and very low birth weight infants and can be even associated with outbreaks in neonatal intensive care units [2–5]. The yeast is also common in hospitalized adult patients with compromised immunity. *C. parapsilosis* is also notorious for its capacity to adhere to foreign material in patients [6]. Several virulence related attributes of *C. parapsilosis* have been discovered and characterized, and the publication of genomes and development of improved gene targeting techniques are poised to rapidly expand our understanding of the pathobiology of this yeast [7–12].

In *C. parapsilosis* three methods have been applied for introducing foreign DNA into the cell: biolistics, electroporation and the single stranded DNA/Li-acetate/PEG method, also known as chemical transformation [13,14]. In contrast to biolistics and electroporation, chemical transformation requires no special equipment, which makes this technique easy to apply and popular. The current protocol for transforming *C. parapsilosis* relies on methods applied for *Saccharomyces cerevisiae* [13,15]. In brief, *C. parapsilosis* cells are grown until a dedicated density, washed, then suspended in 1x TE/100 mM lithium-acetate (1x TELioAc) mixed with ssDNA, the transforming DNA and 3350 molecular weight polyethylene glycol (PEG_3350_) dissolved in 1x TELioAc (PLATE). After overnight incubation at 30°C, the cells are exposed to high temperature for a short period followed by regeneration and then plating onto the given selective media [11]. Although this approach has been used to generate mutants in large scale in our labroatory, we often struggle to gain sufficient numbers of transformants [16,17]. Our results are in line with observations of other authors who reported that the number of transformants of a distinct *C. parapsilosis* strain lags behind *C. albicans* when chemical transformation is applied, and the efficacy drops rapidly when the size of the foreign DNA increases [18]. A successful transformation requires the generation of at least one mutant strain with the appropriate genetic alteration. However, in order to validate biological function with great confidence, multiple confirmed mutant strains are required. Furthermore, when comparing the efficiency of original and optimized or new techniques or methods, low numbers of transformants hampers adequate statistical analysis. These issues become even more challenging during the introduction of multiple fragments into a cell.

Given the absence of corresponding data in the literature on how to improve the competence of *C. parapsilosis* cells, we sequentially tested numerous parameters and optimized methods using a leucine auxotroph laboratory strain (CPL2) to define a more effective alternative protocol for *C. parapsilosis* transformation. Through experimental testing, we found that the transformation efficiency in CPL2 increased by one to two orders of magnitude depending on the size of the transforming DNA. We also found that competent cells could be frozen for at least two months with only minor loss of competence for transformation, which significantly decreases the overall time required for subsequent transformations. Prototroph isolates were also subjected to the newly developed protocol and the new method significantly enhanced transformant recovery in five of the six isolates tested. Hence, our new transformation process represents an important advance in the study of *C. parapsilosis* biology.

## Materials and methods

### Oligonucleotides used in this study

Oligonucleotides used in this study are listed in Supplementary Table S1.

### Strains used in this study

For transformation optimization, the leucine auxotroph laboratory strain of *Candida parapsilosis* (CPL2) was used (*leu2Δ::FRT/leu2Δ::FRT, his1Δ::FRT/his1Δ::FRT, frt::CdHIS1*. Prototroph isolates CDC317, CLIB214, GA1, CBS 1954, CBS 2211 and CBS 6318 were used to validate the new methodology [19] [7,10,20]. For plasmid construction, genomic DNA of *Candida albicans* SC5314 was utilized. Plasmids were propagated in *Escherichia coli* 2T1.

### Plasmid generation

For this study, the plamids pNRVL-LEU2 (pN-L) and pNRVL-caSAT1 (pN-S) were applied [17]. To gain derivatives of these plasmids encoding the fragments used for transformation, genomic DNA of *Candida albicans* SC5314 was amplified using the primers DifLen4kb_REV/DifLen6kb_REV/DifLen8kb_REV/DifLen10kb_REV along with the universal primer DifLen_FOR. Amplicons and plasmids pN-L and pN-S were digested with *Bss*HII and ligated to gain pN-L-4/6/8/10kbp and pN-S-6/10kbp respectively. The accession numbers are presented in Supplementary Table S1.

### Preparation of the transforming fragment

Plasmids carrying the transforming fragment were isolated using Geneaid^TM^ Midi plasmid kit according to the manufacturer’s instructions. To release the transforming fragments, *Stu*I digestion occurred with overnight incubation, then the fragments were precipitated with isopropanol and washed with 70% ethanol. This procedure results in two fragments, the plasmid backbone and another sequence that is reffered to as the “transforming fragment/cassette” or “recombining sequence” hereafter. Pellets were air dried, dissolved in sterile Lonza^TM^ AccuGENE^TM^ water and assayed on 0.8% (m/V) agarose gel to determine concentration and integrity. Proper concentrations of the transforming fragment were set up in a final volume of 10 µl using sterile Lonza^TM^ AccuGENE^TM^ water.

### Growth conditions

Fungal strains were stored in YPD containing 20% (V/V) glycerol at −80°C and routinely maintained on solid YPD medium containing (1% (m/V) glucose (Biolab), 1% (m/V) peptone (Sigma), 0.5% (m/V) yeast extract (VWR), 2% (m/V) agar (Sigma)) and stored at 4°C. Fungal strains were inoculated in YPD liquid media one day before the transformation, and cultivated overnight in an orbital shaker (~ 150 rpm) at 30°C. Cultivation for the transformation was carried out in YPD media.

When pN-L or its derivatives were applied, transformants were selected on minimal media containing 0.19% (m/V) yeast nitrogen base (Sigma), 2% (m/V) glucose (Biolab), 2% (m/V) agar (Sigma) supplemented with drop out media (without histidine and leucine) (See Supplementary Table S1) and cultivated at 30°C for three days [11]. Upon application of the derivatives of pN-S, selection was performed on YPD solid media containing 200 µg/ml nourseothricin (Jena Bioscience) and plates were incubated at 30°C for two days. All media for maintenance or cultivation of fungal strains were supplemented with 100 unit/ml penicillin/streptomycin (Sigma) solution.

*E. coli* 2T1 strain was cultivated in/on LB media (1% (m/V) NaCl (Biolab), 1% (m/V) tryptone (VWR), 0.5% (m/V) yeast extract (VWR) and additionally 2% (m/V) agar (Sigma) for solid media). For selection, the media was supplemented with kanamycin (Sigma) in a final concentration of 50 µg/ml.

### Solutions

PEG solution of 3350 or 4000 molecular weight (Sigma), 1 M lithium-acetate (Sigma) and 10x TE were freshly prepared on the day of the transformation and autoclaved separately. 10x TE was set up by combining 0.5 M EDTA (Sigma) (pH = 7.5 or pH = 8) and 2 M tris(hydroxymethyl)aminomethane (pH = 8) (Sigma). These solutions were replaced on a three month basis. For the solutions and the washing steps sterile Milli-Q® bidistilled water was applied. Sterilized 10x TE and 1 M lithium-acetate was used to prepare 1x TELioAc (1x TE, 0.1 M lithium-acetate). PLATE solution was prepared by combining 50 or 55% (m/V) PEG_3350_ or PEG_4000_, 10x TE and 1 M lithium-acetate in a final concentration of 1x TE and 0.1 M lithium-acetate. Sterile dimethyl sulfoxide (DMSO) (Sigma) was mixed with PLATE solutions in a ratio of 1:10 to generate +DMSO/PLATE. Transforming fragments were dissolved in Lonza^TM^ AccuGENE^TM^ water.

### Transformation

The experimental setup involved the recently described pNRVL-LEU2 (pN-L) and pNRVL-CaSAT1 (pN-S) and their derivatives. These plasmids encode a replacement cassette, that can be released by *Stu*I digestion and targets the intergenic region CpNEUT5L [17]. In the case of pN-L, the transforming cassette encodes only the *LEU2* gene and regulator sequences from *Candida maltosa* (*CmLEU2*) flanked by the target sequences, which complements the leucine auxotrophy of *C. parapsilosis leu2^−/-^* (CPL2) laboratory strain. We chose this small fragment for the optimization (3,214 bp) to reduce the potential bottlenecks of the transformation, and, additionally, using an auxotrophy complemention approach rather than a dominant selectable marker provides other advantages in terms of expense and practicality, since it requires less time for regeneration after heat shock. To test the protocol in prototrophic isolates, derivatives of pN-S were generated carrying *CaSAT1*, which confers resistance against the antibiotic nourseothricin. Characterization of the transformation efficiency was achieved by generating derivatives of pN-L and pN-S encoding 4; 6; 8 and 10 kbp long transforming cassettes.

The optimization procedure was implemented based on the protocol of Holland *et al*. [11] (See original protocol in Supplemental material 1). All the experiments were performed in triplicates. In our first experiment an overnight culture of CPL2 was used to set the initial density to OD_600_ 0.1 and cells were cultivated until OD_600_ 0.7; 1; 1.5 or 2 were achieved prior to harvesting. Competent cell preparation was managed by collecting and washing the cells with half a volume of sterile bidistilled water at room temperature (2000 x g, 5 min). Pellets were washed with 1 ml 1x TELioAc (17.000 x g, 1 min), suspended in 1 ml 1x TELioAc and incubated at 30°C for 30 minutes. Then, samples were divided in half into 1x TELioAc containing either dithiothreitol (DTT) (Sigma) in a final concentration of 7% (V/V) or distilled water instead of DTT (as a control). Samples were incubated with shaking (~150 rpm) for 30 minutes at 30°C. Cells were collected and washed with 1 ml 1x TELioAc (17.000 x g, 1 min), suspended in 200–200 µl 1x TELioAc, and 50–50 µl of competent cells were mixed with 100 µg boiled then ice cooled salmon sperm DNA (10 µl) and 900 ng of the 3,214 bp *CmLEU2* fragment (10 µl). The transformation mixture was incubated for 30 minutes at 30°C without shaking and then gently mixed with PLATE solution prepared with PEG_3350_ or PEG_4000_ and supplemented with 9% (V/V) DMSO or water and incubated overnight. After heat shock (44 °C, 15 min), cells were collected, washed with 950 µl YPD (17.000 x g, 1 min), suspended in 300 µl YPD and shaken (~150 rpm) at 30°C. After two hours, the samples were spun down and placed on ice for 10 minutes to avoid further cell division, and then plated onto selective media.

The 3,214 bp pN-L fragment was used in the following experiments, unless otherwise stated. The effect of dilution was investigated by setting the OD_600_ of CPL2 to 0.1 and cultivating the cells to OD_600_ 0.7; 1; 1.5; 2 (~150 rpm) at 30°C. When cultures reached the dedicated densities, they were either diluted to 0.7 before harvesting or left undiluted to compare the effect of dilution on competence. The cells were washed, suspended in 1x TELioAc, then mixed with the ssDNA and the transforming DNA, and then incubated for 30 minutes at 30°C. PLATE(3350) solution with either 9% (V/V) DMSO or with the same amount of distilled water was added and gently mixed, all other steps were performed as described before.

The effect of the length of the 1x TELioAc exposure was characterized by cultivating the cells from OD_600_ 0.1 to 1.34 in YPD, then 2.73 ml of culture/transformation was used. The cells were collected and washed with distilled water and 1x TELioAc. Competent cells were incubated for 0, 10, 30, 60 or 120 minutes in 1x TELioAc at 30°C without shaking. Compentent cells were added to ssDNA/transforming DNA and left for 30 minutes at 30°C without shaking, and then gently mixed with PLATE(3350) supplemented with 9% (V/V) DMSO (+DMSO/PLATE(3350)). The remainder of the steps were not changed. Competent cells were cultivated and harvested as described above, but cells were immediately mixed with ssDNA/transforming DNA and incubated for 0, 10, 30, 60 or 120 minutes at 30°C without shaking before addition of +DMSO/PLATE(3350) solution. The rest of the steps were unchanged. To define the optimal amount of ssDNA, a twofold dilution series ranging from 0–32 µg/reaction was prepared from boiled then ice cooled salmon sperm DNA. Cultivation and all other steps were performed as described above except that +DMSO/PLATE(3350) solution was added immediately after mixing competent cells with ssDNA/transforming DNA. OD_600_ of the starting cultures were set to 0.05, 0.1, 0.2 or 0.3 to determine optimal initial cell density. All other steps were performed as described above. In subsequent experiments, the starting OD_600_ was set to 0.05. A twofold dilution series was prepared using the *Stu*I digested pN-L fragment ranging from 112 ng (53 fmol) to 7,200 ng (3,392 fmol) to characterize how the transformation efficiency depends on the amount of the transforming DNA, and other parameters were left unchanged. The size dependency of the DNA uptake was investigated by using fragments of 3.2; 4; 6; 8 or 10 kbp, whose concentration was either 900 ng/reaction or normalized to 424 fmol/reaction as 900 ng of the 3.214 bp fragment equals to 424 fmol therefore this latter dimension preserves the CFU:DNA ratio independently of the fragment size. The competence was investigated over a wide range regarding the fragment size and concentration by applying a twofold dilution series of 4; 6; 8 or 10 kbp fragments from 53 fmol to 3392 fmol/reaction. All the other steps were performed as described above. To test the necessity of keeping the cells cooled during preparation, cultures were divided in half after cultivation. One of them was processed according to our optimized protocol (at room temperature) and the other was handled at 4°C (centrifugation) with ice-cold water and 1x TELioAc throughout the whole process until the addition of +DMSO/PLATE(3350), after which all steps were left unchanged. To test how the presence of +DMSO-PLATE(3350) solution influences the uptake of the transforming DNA, the transformation reactions generated as described above were exposed to heat shock immediately and 3; 6; 9; 12; 15 hours after addition of +DMSO/PLATE(3350). For optimizing the length and temperature of heat shock *C. parapsilosis*, cells were transformed with *Stu*I digested pN-L as above with 15 hours of incubation in +DMSO/PLATE(3350) and then were placed into a waterbath set to 40, 42, 44, 46 or 48°C for 6, 9, 12, 15, 18 or 21 minutes.

To confirm the efficiency of the modified protocol over the original recipe, S*tuI* digested pN-L-6, pN-L-10 and pN-S-6, pN-S-10 were used to transform the CPL2 strain and six prototroph isolates of *C. parapsilosis*, respectively, and a no DNA setup was applied as a control. Please note that, as *CaSAT1* selectable marker is shorter than *CmLEU2*, consequently the sizes of the fragments with the dominant selectable marker are smaller as well, 5,657 bp and 9,677 bp respectively. The amount of the recombining sequence was 1–1 µg/transformation.

For all transformation experiments, three individual experiments were performed with two statistical parallels per experiment.

### Storage of competent cells

Two methods were tested for depositing chemically competent *C. parapsilosis* cells. The first one was based on the experiences with *S. cerevisiae* and utilized 5% (V/V) DMSO (Sigma) and 10% (V/V) glycerol (Sigma), whereas the second was developed for *Schizosaccharomyces pombe* and used 30% (V/V) glycerol (Sigma) alone [21,22]. The 10 kbp fragment with the *CmLEU2* marker was used to transform the CPL2 strain. A large batch of competent cells was prepared according to our protocol until the step for adding the cells suspended in 1x TELioAc to the ssDNA/transforming DNA mixture. Instead, the suspension was divided in two, collected and suspended in the two different freezing mediums. 50–50 µl aliquots of the two suspensions were pipetted into microcentrifuge tubes and placed into either a styrofoam box lined and covered with multiple layers of paper towels to provide additional insulation [21] or were put directly into a cardboard microcentrifuge box, covered with two layers of textile towel [22]. Both boxes were placed directly into and stored in a − 80°C freezer. For testing on multiple days, several microcentrifuge tubes were prepared from both setups for assessing transformation and viability. As a control, aliquots before freezing were spread onto non selective media or transformed. Frozen samples were thawed after one day or 2, 4, 6 and 8 weeks. Vials were placed into a 30°C waterbath for 15 sec, then gently flicked and placed back in the waterbath for another 15 sec. Cells were gently spun down and added to the ssDNA/transforming DNA mixture. The rest of the steps were performed according to our modified protocol.

### Software

Heatmaps were generated with PermutMatrix v1.9.3 [23], sequences were edited with ApE – A plasmid Editor v2.0.51, pictures were composed with GNU Image Manipulation Program v2.8.18. Statistical analyses, colony number prediction and curve generation (for Dilution experiment) were performed with GraphPad PRISM v6.01. Data is presented as mean and SEM. Statistical significance was determined by unpaired t tests with Welch’s correction (* p < 0.05; ** p < 0.01; *** p < 0.001; **** p < 0.0001). Colony numbers and significance are summarized in Supplementary Table S2-14.

## Results

### Effect of OD_600_, dimethyl sulfoxide (DMSO), dithiothreitol (DTT) and polyethylene-glycol (PEG)

First, we tested how the optical density of *C. parapsilosis* cultures affects the transformation efficiency, since the available protocols for *Candida* transformation differ in the OD_600_ values prior to harvesting [11,24,25]. We examined OD_600_ ranging from 0.7 to 2.0. As PEG was essential for chemical transformation in related yeast species [26] and work in *Candida* species have utilized 3350 and 4000 molecular weight of PEG [27,28], we tested PEG_3350_ and PEG_4000_ in our experiments. We also tested how the presence or absence of DMSO, a polar aprotic solvent, applied before heat shock alters the efficiency of transformation as the literature indicates that the solvent may increase DNA uptake by *S. cerevisiae* and *C. albicans* [28–30]. Additionally, we also tested if pretreatment with dithiothreitol (DTT), a reducing agent, alters transformation efficiency.

**Figure 1.**
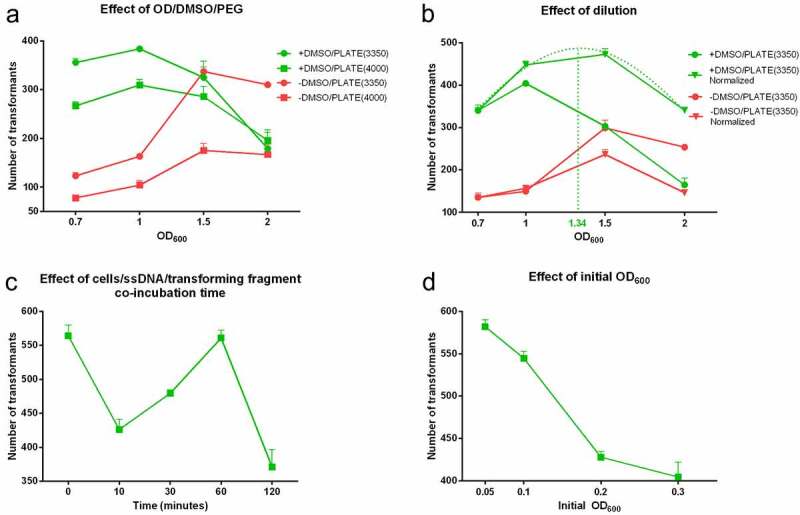
Dependency of the transformation efficiency on the cell density and competent cell preparation parameters. 900 ng of a replacement cassette of 3,214 bp was applied in each reaction. Cells were cultivated in YPD until OD_600_ 0.7; 1; 1.5; 2, collected and mixed with PLATE solutions prepared from PEG_3350_ (PLATE(3350)) (circles) or PEG_4000_ (PLATE(4000)) (rectangles) with or without 9% (V/V) DMSO (green and red symbols respectively). (Panel A). Cells were grown under the same conditions but density was normalized to either OD_600_ 0.7 (triangles) or left undiluted (circles) and competent cells were mixed with PLATE(3350) with 9% (V/V) DMSO (green curves) or distilled water instead (red curves). The optimum was calculated and determined as 1.34 (dotted curve and vertical line) according to GraphPad Prism software (Panel B). Cells were prepared according to the optimized parameters and cells suspended in 1x TELioAc were immediately mixed with ssDNA/transforming DNA then incubated over a range of 0–120 minutes (Panel C). The initial cell concentration was set to four different values ranging from OD_600_ 0.05 to 0.3. Competent cells were prepared as optimized so far (Panel D). Data are presented as the average and SEM of three independent biological parallels

DTT had an adverse effect on the DNA uptake irrespectively of the rest of the parameters (Supplementary Figure S1). Figure 1a demonstrates the numbers of transformants recovered under the different conditions tested in the absence of DTT. In the presence of DMSO the peak of the curves was at OD_600_ of 1, while in its absence the peak occurred at an OD_600_ of 1.5. In the presence of DMSO, the transformation sequentially declined from OD_600_ 1 to 1.5 to 2. In contrast, when DMSO was omitted, OD_600_ 1.5 and 2 conditions significantly outperformed the lower cell densities except for PEG_4000_ at OD_600_ of 2, where no significant difference was established. When DMSO was applied, the addition of PEG_3350_ led to the recovery of more transformants compared to PEG_4000_ at OD_600_ 0.7 and 1, while at OD_600_ of 1.5 and 2 the two PEG conditions were similar (p = 0.39 and p = 0.70, respectively). Without DMSO, PEG_3350_ produced significantly more transformants compared to PEG_4000,_ and the numbers at an OD_600_ 1.5 were similar to that of DMSO medium with PEG_3350_ (p = 0.76).

### Dilution

Since the cell numbers in Figure 1a were aliquots directly from cultures with different OD_600_, variations in transformation efficiency could be directly releated to cell density or due to differences in cell structures and replication rates of the cells of various ages. To explore this issue, we normalized the cell numbers in OD_600_ 1, 1.5 and 2 to OD_600_ 0.7. Since PEG_3350_ outperformed PEG_4000_, PEG_3350_ was used with or without DMSO and without DTT pretreatment. Figure 1b shows the numbers of transformants with or without DMSO under conditions where cell numbers were normalized to OD_600_ 0.7. The presence of DMSO with normalized cell densities produced the highest numbers of transformants and resulted in the normalized OD_600_ 1.5 outperforming the other conditions, althought it was not significantly different from OD_600_ 1 (472.7 ± 13.6 vs 448.0 ± 8.5; p = 0.21). The absence of DMSO similarly resulted in OD_600_ 1.5 producing the highest numbers of transformants (299.0 ± 18.9).

For the normalized densities in the presence of DMSO, the number of transformants followed a Gaussian-like distribution in terms of OD_600_ dependency. The built in „Curve generation” feature of Prism software allowed us to fit a curve onto this graph to determine the highest point of this curve (referring the most transformants) and the OD_600_ value where this could be achieved. This was established at OD_600_ 1.34, where the calculated number of colonies was approximately 487. According to this, we harvested the cells at OD_600_ 1.34 ± 0.05 in the remaining experiments and used a PLATE(3350) solution containing 9% (V/V) DMSO (+DMSO/PLATE(3350)). We also took into account the „dilution factor”. The initial volume of the cultures used for transformation was 45 ml, which was sufficient for eight transformations. Thus, for a suspension of OD_600_ 0.7 using a culture of OD_600_ 1.34, the YPD:suspension target ratio was 515:485, which represents 2.73 ml of the original culture for one transformation reaction. To avoid dilutions in the rest of the experiments for this study, we used 2.73 ml/transformation from the culture of OD_600_ 1.34 ± 0.05 suspension. We scaled down the first washing step accordingly, but otherwise followed the rest of the steps.

### TELioAc and PLATE incubation

The duration of exposure to lithium-acetate can significantly impact transformation efficiency in yeast [28]. Hence, we investigated if the length of TELioAc exposure and the incubation of cells with ssDNA and the transforming fragment altered DNA uptake. Transformants were achieved with exposures to 1x TELioAc for 0, 10, 30, 60 or 120 minutes while keeping other steps of the process unchanged (Supplementary Figure S2). We found that immediate addition of the cell suspension to the ssDNA/transforming DNA mixture yielded the most colonies (502.7 ± 14.4) that was in line with the protocol of Holland and coworkers [11]. Interestingly, a 60 minute exposure produced the second highest, but significantly lower yield (421.3 ± 11.22; p = 0.0129). We next assessed the effects of incubation duration with ssDNA/transforming fragments. Figure 1c shows the recovery of transformants using the time 0 1x TELioAc mixture incubated with the ssDNA/transforming fragments for 0, 10, 30, 60 or 120 minutes. Interestingly, the shape of the curve was very similar to that achieved with 1x TELioAc. Immediate combination of the 1x TELioAc and ssDNA/transforming fragments with the +DMSO/PLATE(3350) resulted in the most transformants (564.0 ± 15.6) though the recovery efficiency achieved with a 60 minute incubation was similar (560.7 ± 11.6; p = 0.87). Hence, we found that the immediate mixture of competent cells with the transforming DNA/ssDNA solution followed by the immediate addition to +DMSO/PLATE(3350) was the simplest and most effective method for transforming *C. parapsilosis*.

### Single stranded DNA

Single stranded carrier DNA (ssDNA) is introduced to competent cells to 1) mask the charge of the fungal cell wall and aid the delivery of the transforming DNA into the cytoplasm and 2) oversaturate endogenous nucleases of the cell in order to avoid degradation of the transforming DNA [15,31]. Thus, we investigated if the quantity of ssDNA applied altered the transformation efficiency.

The transformation efficiency was dependent on the amount of the ssDNA and that the increased doses assessed resulted in a saturation curve in the range of 0 to 320 µg/reaction (Supplementary Figure S3). In the absence of ssDNA, we generated 149.3 ± 6.6 colonies. The highest number of transformants recovered occurred with 320 µg salmon sperm DNA (537.3 ± 7.1), but this was similar to that achieved with 160 µg (526.0 ± 3.5; p = 0.25). We used Prism for “Curve generation” and determined that the average number of the transformants at 100 µg of salmon sperm DNA would be ~510 colonies. To increase the number of the transformants from ~510 (100 µg carrier DNA) to ~537 (320 µg carrier DNA) would require a 3.2-fold increment in the amount of the ssDNA, while it leads to only 5.3% rise in the transformation efficiency. Thus, we decided the salmon sperm DNA 100 µg/reaction as suggested in the protocol of Holland and coworkers [11] was appropriate for our methodology.

### Initial OD

The protocols in the literature for chemical transformation of *Candida* species differ in the value where the absorbance of the initial suspension should be set [11,25]. Figure 1d details the impact of the initial OD_600_ on transformation efficiency depends on the initial OD_600_. The efficiency of DNA uptake was greatest at OD_600_ 0.05 (582.0 ± 8.1) and the numbers of recovered transformants decreased significantly (p < 0.05) as the OD_600_ increased. Compared to OD_600_ 0.05, the increased densities were 6.4%, 26.5% and 30.5% lower, respectively. Therefore, we identified OD_600_ of 0.05 in most appropriate density for *C. parapsilosis* transformation.

### DNA amount and size

**Figure 2.**
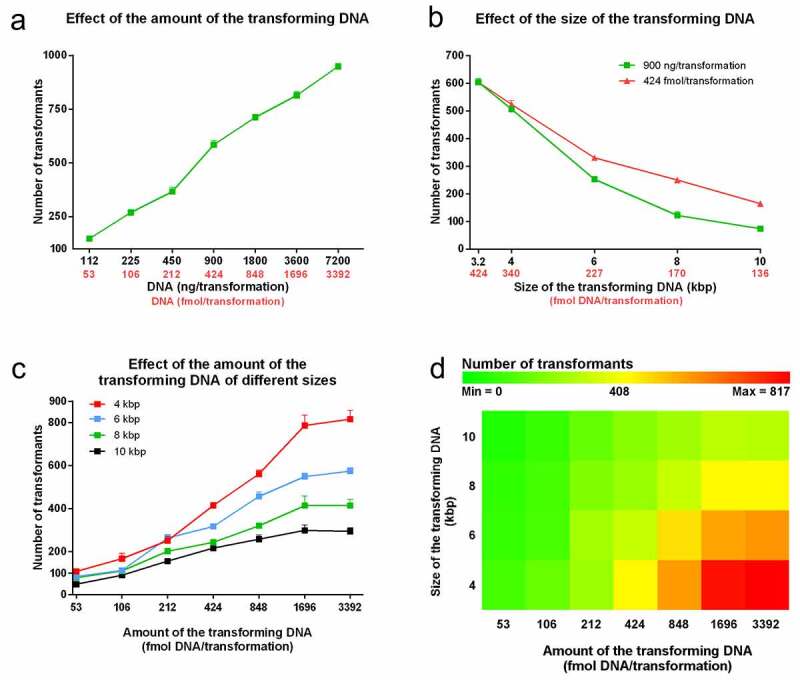
Dependency of the transformation efficiency on the size and the amount of the transforming DNA. Competent cells were prepared according to the optimized steps of the protocol. A range of 112 ng (53 fmol) to 7,200 ng (3,392 fmol) of the 3,214 bp long fragment was applied in twofold dilutions in each transformation (Panel A). Plasmids encoding 4; 6; 8 and 10 kbp replacement cassettes were generated and used with the 3,214 kbp long fragment. Transformations were carried out with 900 ng as well as 424 fmol of these sequences (Panel B). As a comprehensive analysis on the effect of size, the 4; 6; 8 and 10 kbp cassettes were applied in twofold dilutions from 3,392 fmol to 53 fmol/reaction (Panel C and D). All data points represent means of three independent transformations with SEM, except Panel D where the heat map was generated using the averages of the three independent experiments at each point

It is a general observation that more DNA leads to more transformants. Figure 2a characterizes how *C. parapsilosis* transformation efficiency depends on the amount of the transforming DNA when the 3,214 bp fragment is applied. We found that the number of the transformants approximately fit on a line, such that doubling the amount of the transforming DNA results in a significant increase in the number of the transformants by nearly the same extent (+133.6 ± 18.0 transformants/duplication). The number of transformants ranged from 148.0 ± 10.6 using 112 ng/53 fmol to 949.3 ± 10.4 with 7.2 µg/3,392 fmol.

We utilized the *Stu*I digested fragment of pN-L because of its small size, but this does not represent the typical size of a transforming DNA. To investigate how the transformation efficiency depends on the size of the DNA, besides pN-L, we included four of its derivatives; pN-L-4kpb, pN-L-6kbp, pN-L-8kbp and pN-L-10kbp that once digested with *Stu*I, provide a 4,014, 5,992, 8,006 and 10,012 bp long fragment respectively. These fragments carry exogenous sequences from *C. albicans* not coding any known ORFs that 1) minimizes the possibility of an unwanted recombination and 2) avoids the effect of a foreign gene’s expression on the viability of the transformants. Besides 900 ng, also 424 fmol DNA/reaction was applied to keep the DNA:CFU ratio fixed in all the setups. Figure 2b shows that increasing the size of the transforming DNA resulted in a decrease in the transformation efficiency independently of whether it was measured in ng or fmol. Keeping the DNA:CFU ratio constant yielded more colonies, which represented a higher mass of DNA as the length of the fragment increased. The number of the transformants recovered using 4 kbp fragment was 506.7 ± 8.7 (ng condition) and 524.0 ± 14.1 (fmol condition); p = 0.36. However, as the size (and therefore the amount measured in ng) of the transforming cassette increased, the differences became significant. For example, 900 ng of the 6 kbp fragment (227 fmol) resulted in 252.7 ± 8.7 colonies while 424 fmol yielded 330.7 ± 7.9 colonies; p = 0.0027. In the case of the 8 kbp fragment, the average number of the colonies increased two-fold from 122.0 ± 13.5 (900 ng/170 fmol) to 250.0 ± 7.2 (424 fmol); p = 0.0034.

Next we recorded how the fragments of different sizes influence the DNA uptake when applied in various amounts (Figure 2c). We found that as the amount of the transforming DNA increased, the efficiency of transformation was greater with the smaller fragments, reaching a maximum with 817.0 ± 40.3 colonies when the 4 kbp fragment was used. We also noticed that, in contrast to what we observed using the 3,214 fragment, these curves follow a saturation shape for the range over which the fragments were applied. As the length of the fragments increased the saturation occurred at lower amounts of DNA. With the maximum number of transformants related to every given fragment size in mind, we calculated the „maximum effective amount” of the transforming DNA, where the further increment did not result in significantly more transformants. This was determined as 1,696 fmol (787.0 ± 48.5 colonies), 1,696 fmol (549.0 ± 16.7 colonies), 848 fmol (320.7 ± 12.4 colonies) and 424 fmol (217.0 ± 20.2 colonies) for the 4, 6, 8 and 10 kbp fragments, respectively.

### Effect of ice

**Figure 3.**
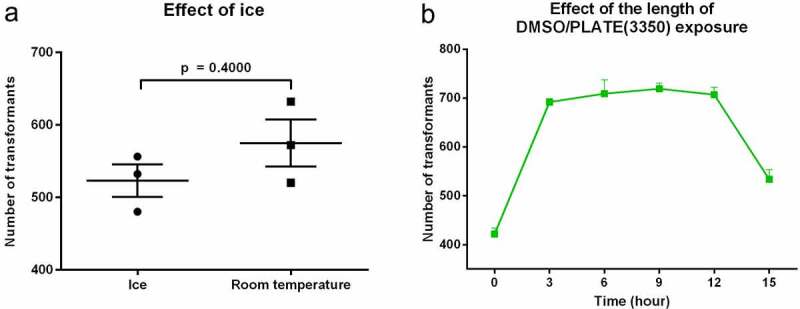
Dependency of the transformation efficiency on the temperature of the competent cell preparation and the length of +DMSO/PLATE treatment. Cells were kept either on ice or at room temperature upon competent cell preparation (Panel A). Effect of the length of competent cells/ssDNA/transforming DNA incubation along with +DMSO/PLATE(3350) was tested over a range of 0–15 hours (Panel B). All experiments were performed in triplicates. Data are presented as mean and SEM.Significance was calculated with unpaired t test with Welch’s correction

Current yeast protocol recommends keeping cells on ice from harvesting until the addition to the carrier and transforming DNA [11]. Figure 3a reveals that the average number of transformants was actually slightly higher when competent cells were prepared at room temperature (574.7 ± 32.4) compared to maintaining the cells on ice (522.7 ± 22.4), but this difference was not significant (p = 0.27). Thus, we proposed maintaining the cells at room temperature for *C. parapsilosis* transformations.

### PLATE induction

Figure 3b reveals the impact of exposing transformation competent cells to DMSO on the efficiency of the transformation. Immediate application of heat shock right after the addition of +DMSO/PLATE(3350) to the transformation competent cells resulted in an average of 421.7 ± 12.2 transformants. However, long incubations prior to heat shock significantly increased the uptake of the transforming DNA. Interestingly the transformation efficiency was similar from 3 to 12 hours (692.0 ± 7.2 at 3 hours, 709.3 ± 28. 5 at 6 hours, 719.3 ± 11.6 at 9 hours and 707.3 ± 14.6 at 12 hours). The number of transformants at 15 hours, 534.0 ± 19.6, was significantly greater than time 0 (p = 0.0128), but also significantly lower than the other times evaluated (p < 0.01), after which it decreased at 15 hours. The differences between 3 (692.0 ± 7.2); 6 (709.3 ± 28. 5); 9 (719.3 ± 11.6) and 12 (707.3 ± 14.6) hour incubations were not significant from each other in any comparison. However, considering the length of the competent cell preparation, regeneration period and plating, the times between 3 and 12 hours did not meet the regular daily routine of the laboratory; therefore, we selected the 15 hour incubation durations for all the upcoming experiments.

### Length and temperature of heat shock

The effect of the length and temperature of heat shock on the recovery of *C. parapsilosis* transformants was investigated in a range of 6–21 min and 40–48°C (Supplementary Figure S4A and B). Interestingly, transformants were identified in the absence of heat shock (123.0 ± 13.8). We found that heat shock at 44°C most consistently produced the highest numbers of transformants. The current standard of 15 minutes incubation at 44°C produced the maximum number (641.3 ± 11.6) of the transformants at this temperature, which was not significantly different from the maximal number of transformants identified at 9 minutes at 46°C (656.0 ± 21.1; p = 0.58). Incubation at temperatures below 44°C resulted in significantly lower numbers of transformants except at 21 minutes where the recovered colonies were similar between 40, 42 and 44°C (p > 0.05). Transformant numbers significantly declined over time with incubations at 48°C. The results supported the continued use of heat shock at 44°C for 15 minutes.

### Comparison of the protocols

**Figure 4.**
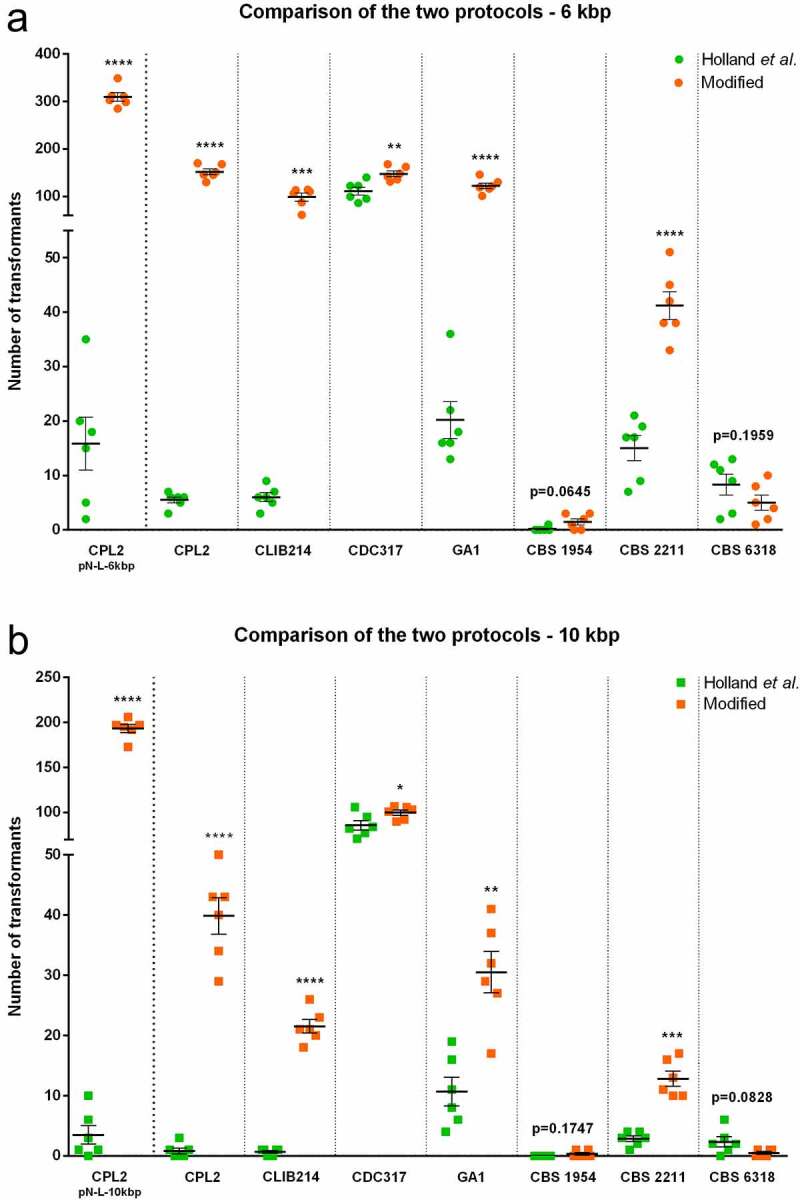
Comparison of the two protocols. One µg of the 6 kbp (5,657 bp) (Panel A) or 10 kbp (9,677 bp) (Panel B) fragments were applied carrying the dominant selectable marker *CaSAT1*. CPL2 transformed with pNRVL-LEU2-6/10 derivatives were applied as references. All experiments were performed in three biological parallels with two statistical parallels. Data are presented as mean and SEM. Significance was calculated with unpaired t test with Welch’s correction (* p < 0.05; ** p < 0.01; *** p < 0.001; **** p < 0.0001)

In order to assess the overall efficiency of our modified protocol compared to that published by Holland *et al*. [11], we subjected the auxotroph strain CPL2 and prototroph isolates to both methods. Figure 4 demonstrates the outcomes between the methods. In the case of the CPL2 laboratory strain, the modified protocol resulted in one and two orders of magnitude more prototroph transformants than the reference method depending on the size of the fragment, with 309.8 ± 8.8 versus 15.8 ± 4.8 (6 kbp; p < 0.0001) and 193.3 ± 4.5 versus 3.5 ± 1.6 (10 kbp; p < 0.0001). This difference was also apparent when the fragment encoding the dominant selectable marker was applied to alter the same auxotroph strain, where the modified protocol resulted in 151.7 ± 6.2 transformants compated to 5.5 ± 0.6 (6 kbp; p < 0.0001) and 39.8 ± 3.0 versus 0.8 ± 0.5 (10 kbp; p < 0.0001) colonies. The performance of the modified method was similar when the CLIB214 isolate, the parental strain of CPL2, was transformed as the modified protocol produced 98.5 ± 8.5 and 21.50 ± 1.1 colonies while the Holland protocol resulted in 6.000 ± 0.8165 and 0.6667 ± 0.2108 colonies when the 6 (p < 0.001) and the 10 kbp (p < 0.0001) fragments were used, respectively. The modified protocol produced significantly more transformants with the clinical isolates CDC317 and GA1 (p < 0.05). Although the environmental isolate CBS 2211 produced significantly more colonies with the modified protocol with both fragment sizes (p < 0.001), isolates CBS 1954 and CBS 6318 produced similarly low (~less than 10 colonies) numbers of transformants with both protocols. Increasing the amount of transforming DNA from 1 to 5 µg/transformation significantly increased the colony yield for CBS 1954 using the modified protocol (p < 0.0001 for 6 kpb fragment and p < 0.05 for 10 kpb), although the total colonies were less than 30 (Supplementary Figure S5). In contrast, CBS 6318 transformants significantly increased using the Holland protocol when applying either transcript length (p < 0.0001).

### Depositing

The transformation protocol takes a total of three days: day 1) inoculation; day 2) cultivation, competent cell preparation and setting up the transformation; day 3) heat shock and plating. A goal of our approach to modifying the methodology for *C. parapsilosis* was to shorten the time required for transformation, which we aimed to achieve by depositing competent cells for subsequent use. The cells were transformed with the 10 kb fragment that was the most challenging to manage as demonstrated in the previous experiments.

**Figure 5.**
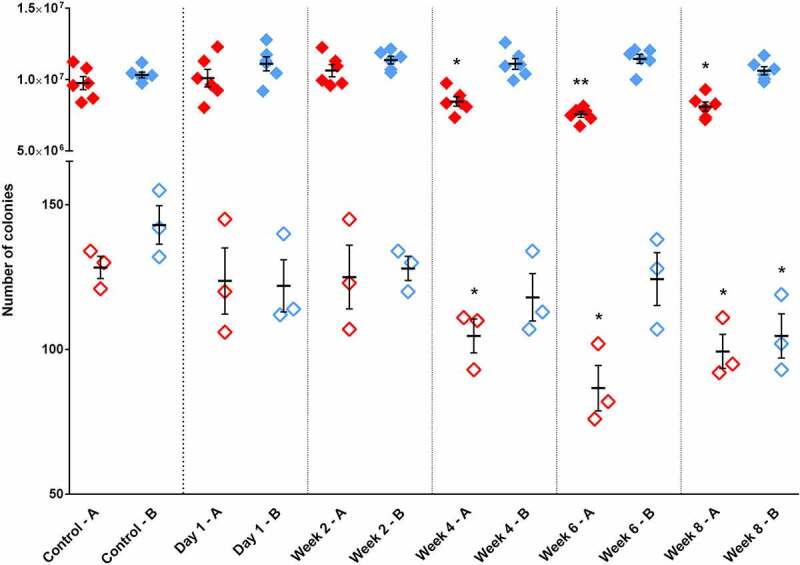
Investigation of the viability and competence of deposited competent cells. Cells of CPL2 strain were prepared according to the modified protocol and suspended in two different freezing solutions. A) containing 5% (V/V) DMSO and 10% (V/V) glycerol in 1x TELioAc (red symbols) and B) 30% (V/V) glycerol in 1x TELioAc (blue symbols). Three tubes were taken for viability test (solid symbols) and three for transformation with 1 µg of pN-L-10 fragment (empty symbols) on the day of the competent cell preparation (before freezing) and 1 day, 2; 4; 6 or 8 weeks after freezing. Significant drop in the viability or the competence was calculated with unpaired t test with Welch’s correction in comparison with the respective control conditions (* p < 0.05; ** p < 0.01)

Figure 5 presents the viability and capacity for transformation of *C. parapsilosis* competent cells stored under two conditions for one day to eight weeks. The viability of yeast cells stored in freezing solutions comprised of 5% (V/V) DMSO and 10% (V/V) glycerol in 1x TELioAc decreased significantly by week 4 of freezing, changing from 9.8 × 10^6^ ± 4.6 x10^5^ at time 0 to 8.5 × 10^6^ ± 3.3x10^5^ (p < 0.05). Similarly, the number of transformants drecreased from 128.3 ± 3.8 to 104.7 ± 5.8 (p < 0.05) over the 4 weeks. In contrast, cells stored in 30% (V/V) glycerol in 1x TELioAc freezing medium had similar viability over eight weeks. However, the competence decreased as the number of transformants gradually decreased from 143.0 ± 6.7 to reach a significant reduction in recovery at week 8 to 104.7 ± 7.6 colonies (p < 0.05). According to our findings we recommend storage in 1x TELioAc supplemented with 30% (V/V) glycerol and using the competent cells within 8 weeks (See Supplementary material 2).

### Modified protocol description

Reagents

Glucose (Biolab)

Peptone (Sigma)

Yeast extract (VWR)

Agar (Sigma)Distilled water for liquid/solid media

Nourseothricin (NTC) (for dominant selectable marker) (Jena Bioscence)

Yeast nitrogen base (YNB) (for auxotrophic selectable marker) (Sigma)

10x Drop out solution (for auxotrophic selectable marker) (recipe in Supplementary Table S1)

EDTA-Na2*H2O (Sigma)

Tris(hydroxymethyl)aminomethane (Sigma)

Lithium-acetate (Sigma)

Polyethylene-glycol 3350 (PEG_3350_) (Sigma)

Sterile DMSO (Sigma)

Milli-Q bidistilled water

Salmon sperm DNA (10 mg/ml stock) (Sigma)

37% (m/V) HCl (for pH adjustment) (Molar)

10 M NaOH (for pH adjustment) (Molar)

Transforming DNA

Equipment

1.5 ml microcentrifuge tubes

50 ml conical tubes

Centrifuge for 1.5 ml microcentrifuge tubes

Centrifuge for 50 ml conical tubes

Water bath

Petri plates

Incubator (30°C)

Spectrophotometer with cuvettes

Sterile box

Glass flask for cultivation

Orbital shaker (30°C, 150 rpm)

Ice

Pipettes and tips

Aluminum foil

Arrangements

Prepare YPD liquid media for cultivation: 1% (m/V) glucose, 1% (m/V) peptone, 0.5% (m/V) yeast extract and autoclave.

Prepare 1.) minimal plates: 0.19% (m/V) yeast nitrogen base, 2% (m/V) glucose, 2% (m/V) agar and 1/10 volume of 10x Drop out solution after autoclave, or 2.) YPD-NTC plates: YPD liquid media + 2% (m/V) agar, autoclave and add NTC in a final concentration of 200 µg/ml. To prepare 10x TE use 0.5 M EDTA (pH = 7.5) and 2 M tris(hydroxymethyl)aminomethane (pH = 8) stocks solutions. (Replace these solutions on a three month basis.)

Prepare 10x TE, 1 M lithium-acetate, and 55% (m/V) PEG_3350_ freshly on the day of the transformation. Sterilize these solutions and use as stocks to prepare 1x TELioAc (1/10 volume of 1 M lithium-acetate, 1/10 volume of 10x TE and 8/10 volume of water) and +DMSO/PLATE(3350) solution (1/10 volume of 1 M lithium-acetate, 1/10 volume of 10x TE, 8/10 volume of 55% (m/V) PEG_3350_. Mix this solution with sterile DMSO in a ratio of 10:1 and mix vigorously. Protect the mixture from light by covering the tube containing the +DMSO/PLATE(3350) solution with aluminum foil. Use sterile Milli-Q water for the solutions and washing the cells.

Place 10 µl x (number of transformation + 1) salmon sperm DNA in boiling water in a microcentrifuge tube for 10 minutes and then cool it down rapidly on ice (ssDNA) and keep it on ice.

Perform experiment at room temperature unless otherwise stated.

For *C. parapsilosis* cultivation one can scale up the volume, but the volume of the suspension should not exceed 1/3 of the total volume of the flask.

Procedure

1.) Inoculate the strain in 5 ml YPD and incubate overnight at 30°C (~150 rpm)

2.) On the other day adjust OD_600_ to 0.05 in YPD in a 250 or 500 ml glass flask and incubate at 30°C (~150 rpm)

3.) When OD_600_ 1.34 ± 0.05, transfer 2.73 ml suspension/transformation into a 15 or 50 ml falcon tube (depends on the volume)

4.) Centrifuge at ~2000 x g, 5 minutes

5.) Discard supernatant and wash the cells with half a volume of water (~2000 x g, 5 minutes)

(If You took for instance 20 ml of suspension, use 10 ml of water), during this time

6.) Set up transformation mixtures in 1.5 ml microcentrifuge tubes on ice as follows:

10 µl ssDNA (bioled and ice cooled)

10 µl transforming fragment (100 ng/µl)

Mix contents, then spin the tubes down in a bench top centrifuge and keep them on ice

7.) Discard supernatant and suspend the cells in 1 ml 1x TELioAc

8.) Centrifuge in a bench top centrifuge ~17,000 x g, 1 minute

9.) Remove supernatant and suspend the pellet in 50 µl 1x TELioAc/transformation

10.) Add 50 µl of this suspension to the reaction mixture from Step 6.) and mix gently

11.) Add 770 µl freshly prepared DMSO/PLATE(3350) solution immediately, then turn the tubes upside down and flick them gently three times to mix contents

12.) Incubate for 15 hours at 30°C overnight (Static)

13.) On the other day perform heat shock by placing the samples into a water bath (44 °C for 15 minutes)

14.) Centrifuge 17,000 x g, 1 minute (benchtop centrifuge)

15.) Carefully remove as much supernatant as possible

16.) Add 950 µl of YPD without disturbing the pellet, then turn the tubes upside down and flick them gently

17.) Centrifuge at 17,000 x g, for 1 minute (benchtop centrifuge)

18.) Remove as much supernatant as possible and suspend the pellet in 300 µl of YPD

19.) Incubate at 30°C with shaking (~150 rpm) for 2 hours (auxotrophic selectable marker) or for 4 hours (dominant delectable marker)

20.) Centrifuge at 2,400 x g for 3 minutes (benchtop centrifuge)

21.) Remove 150 µl of supernatant

22.) Suspend the pellet in the rest of the supernatant and plate onto selective media

23.) Incubate at 30°C for 3 days (auxotrophic -) or 2 days (dominant selectable marker)

## Conclusions

In this study, we aimed to optimize the steps of the chemical transformation procedure for the global fungal pathogen *C. parapsilosis* in order to simplify and improve the efficiency of genetic interventions. We based our approach on the currently utilized protocol commonly applied for *C. parapsilosis* transformations [11]. Our modifications decrease the number of steps required, provides the ability to utilize frozen compentent yeast, and enables the transformation of even large fragments in this pathogenic fungus.

One important finding was the defining of the optimal cell harvest density together with the best dilution of the identified density. Although OD_600_ of 1 performed the best when undiluted, normalization of cells obtained from OD_600_ of 1.5 cultures to OD_600_ of 0.7 yielded the greatest numbers of transformants. The influence of DMSO in the PLATE solution was not a priori obvious, as we found that it depended on the density of the suspension and also on the molecular weight of the PEG used. This finding might provide an explanation to the controversial observations in the literature regarding the effect of DMSO [28–30]. In our setup, we recorded the best result with the application of +DMSO/PLATE(3350), when cells were grown until OD_600_ 1.34 and 2.73 ml suspension/transformation was applied. We found that cooling the cells during competent cell preparation did not significantly improve transformation efficiency, and the most (or not significantly less) transformants were obtained when the competent cells were mixed with the ssDNA, the transforming fragment and the +DMSO/PLATE(3350) solution as soon as possible. Interestingly ssDNA was not essential to gain transformants (when using 900 ng of the 3,214 bp fragment), but obviously the efficiency increased to more than 3-fold when 100 µg was present, which correlates with other reports in yeast [32].

One of our major goals was to characterize the efficiency of DNA uptake in terms of its size and amount. Although transforming fragments are usually measured in µg, we decided to keep the CFU:DNA ratio constant to provide a more consistent comparison. Independently of the amount applied, there was a negative correlation between the size of the fragments and the number of the transformants. In the case of the 3,214 bp fragment this relationship was linear meanwhile in the range of 4–10 kbp, a plateau formed in every case in terms of the transformation efficiency, which occurred at lower amounts as the fragment size increased. This likely relates to a DNA uptake mechanism delimited by the size of the transforming fragment. This phenomenon might be due to the charge of the surface of the cells as the same amount of fragments (measured in fmol) provide more negative charges as their size increases, which might limit the attachment of further DNA fragments to the surface of the cell wall and, consequently, the transformation efficiency as well. These results provide evidence that the “more DNA results in more transformant” expectation is valid only in a certain range. With this information in mind one can design transformation experiments in a more cost- and time-effective way.

The protocols applied for *C. albicans* and *C. parapsilosis* originate from methods optimized for *S. cerevisiae*. For baker’s yeast, the optimum conditions of heat shock is 42°C for 20 minutes [15]. Notably, Walther and Wendland proposed that this might not be sufficient for *C. albicans*, a species that is highly adapted to the human body temperature [28]. Therefore, we tested the effect of the length and temperature of the heat shock step on the transformation efficiency. Our results confirmed the parameters described in the protocol of Holland and coworkers (44°C, 15 minutes); however, 46°C for 9 minutes also proved to be effective [11]. We surmise that lower temperatures likely fail to provide enough shock to promote DNA uptake, while higher temperatures likely harm the cells.

Testing our modified protocol in comparison with the one of Holland *et al*. with six prototroph isolates showed that the new methodology performed significantly better when either 6 (5,657) or 10 (9,677) kbp fragments were applied in five out of six isolates. The greatest difference was observed between the two protocols when CLIB214 was transformed with 6 and 10 kbp fragments with increases in efficiency of 16.4x and 32.2x, respectively, possibly because the derivative of this strain was used to optimize the procedure. According to our observations, competence itself seems to be a strain dependent feature, indicating that further optimization might be necessary to develop methods for transforming different isolates. However, we also found that one could also simply increase the amount of transforming DNA to generate more transformants.

In summary, our modified method for *C. parapsilosis* transformation increases the efficiency of transformation and permits the use of larger DNA fragments. Moreover, the protocol permits the storage of frozen competent cells that maintain their transformation efficiency for several weeks, reducing the time for multiple genetic manipulation proceedures.

## Supplementary Material

Supplemental Material

## Data Availability

Plasmids generated in this study are available upon request, sequences are uploaded to GenBank. Accession numbers can be found in Supplementary Table S1. https://www.ncbi.nlm.nih.gov/genbank/
