# Supplementary material for: Enhancing the chemical transformation of Candida parapsilosis
Source: Virulence. 2021 Mar 17;12(1):937–50. doi: 10.1080/21505594.2021.1893008 (PMC7993187; doi:10.1080/21505594.2021.1893008)
Supplement: Supplemental Material [file KVIR_A_1893008_SM1043.zip › Supplementary Figures.pdf]

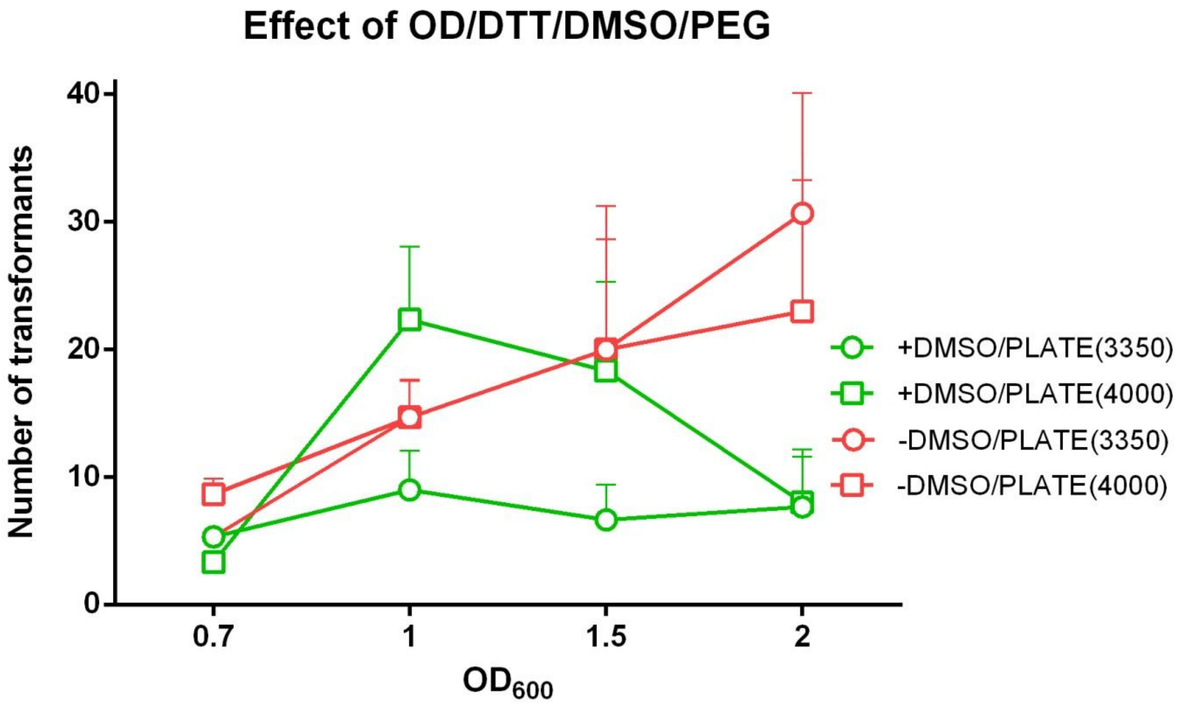

Supplementary Figure S1. Dependency of the transformation efficiency on the cell density and competent cell preparation parameters upon DTT pretreatment. 900 ng of a replacement cassette of 3,214 bp was applied in each reaction. Cells were cultivated in YPD until OD_{600} 0.7; 1; 1.5; 2, collected and incubated in 1x TELioAc in the presence of 7 % (V/V) DTT, and then mixed with PLATE solutions prepared from PEG_{3350} (PLATE(3350)) (circles) or PEG_{4000} (PLATE(4000)) (rectangles) with or without 9 % (V/V) DMSO (green and red symbols respectively). Data are presented as the average and SEM of three independent biological parallels.

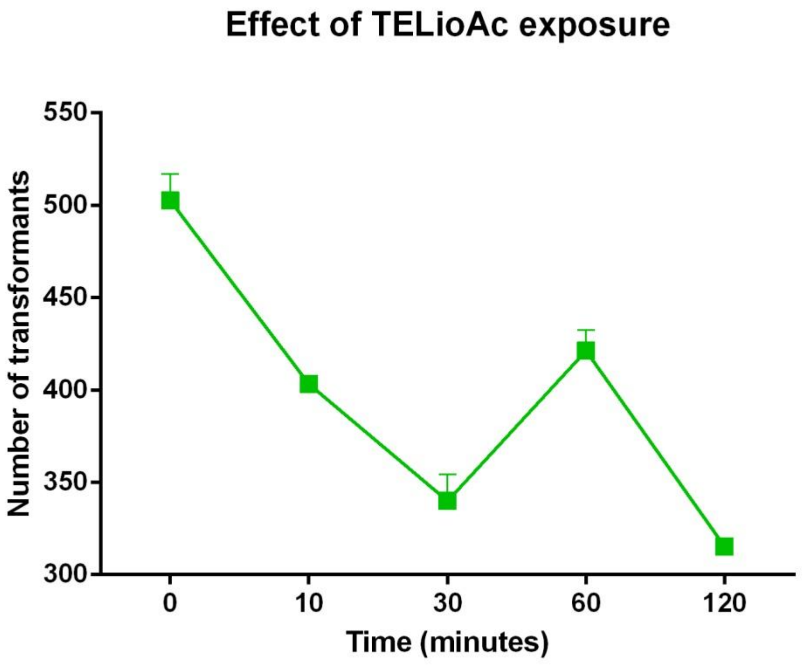

Supplementary Figure S2. Investigation of the effect of the length of the TELioAc exposure on the transformation efficiency. Cells were prepared according to the optimized parameters and the effect of the length of 1x TELioAc exposure was investigated over a range of 0-120 minutes. Data are presented as the average and SEM of three independent biological parallels.

.

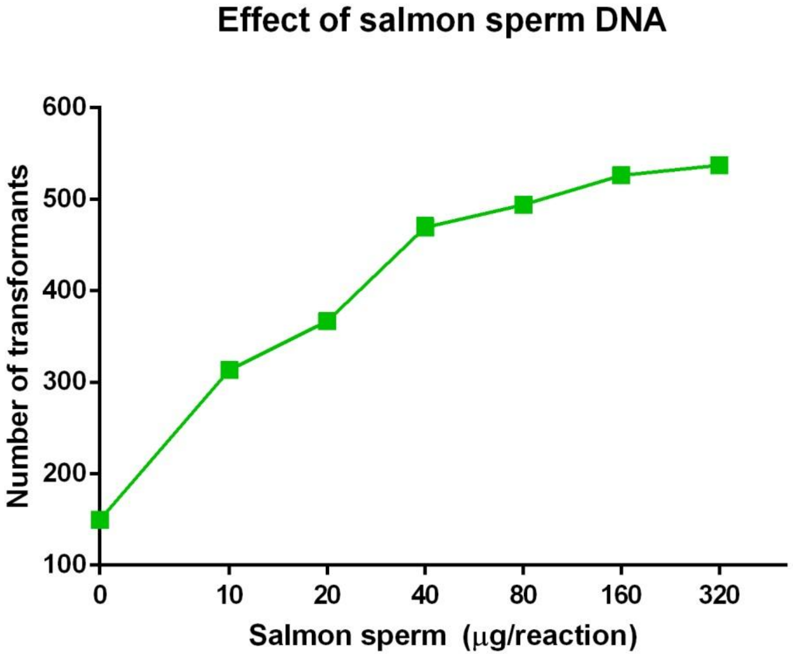

Supplementary Figure S3. Investigation of the effect of salmon sperm DNA on the transformation efficiency. 900 ng of a replacement cassette of 3,214 bp was applied in each reaction. Cells were prepared under the circumstances optimized so far. Salmon sperm DNA was applied in a range of 0-320 μg/reaction. Data are presented as the average and SEM of three independent biological parallels.

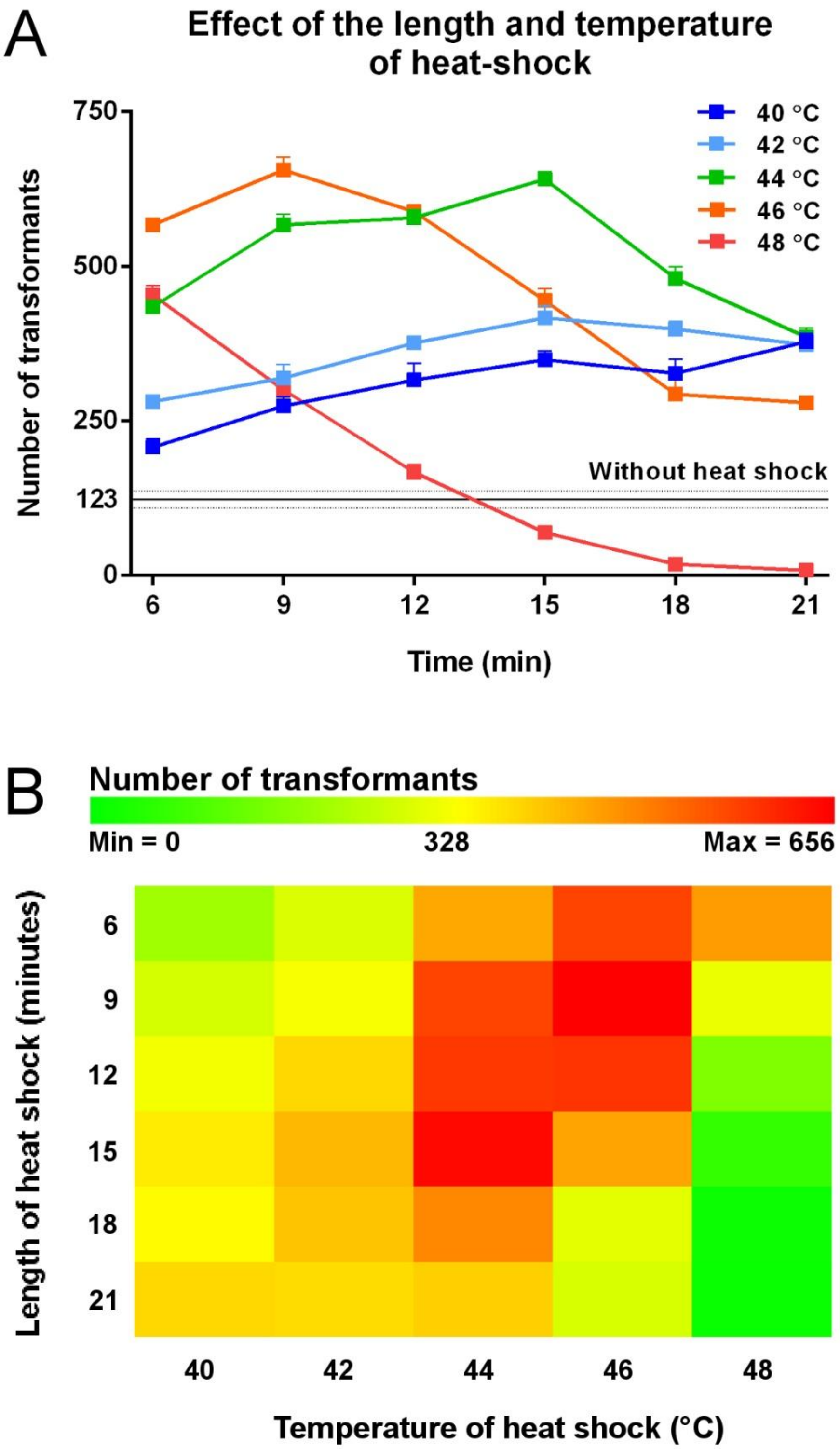

Supplementary Figure S4. Dependency of the transformation efficiency on the duration and extent of heat shock. These parameters were investigated by exposing the cells to 40, 42, 44, 46 or 48 °C for 6; 9; 12; 15; 18 or 21 minutes, a sample without a heat shock was applied as a control. All experiments were performed in triplicates. Data are presented as mean and SEM, except Panel B where the heat map was generated using the averages of the three independent experiments at each point.

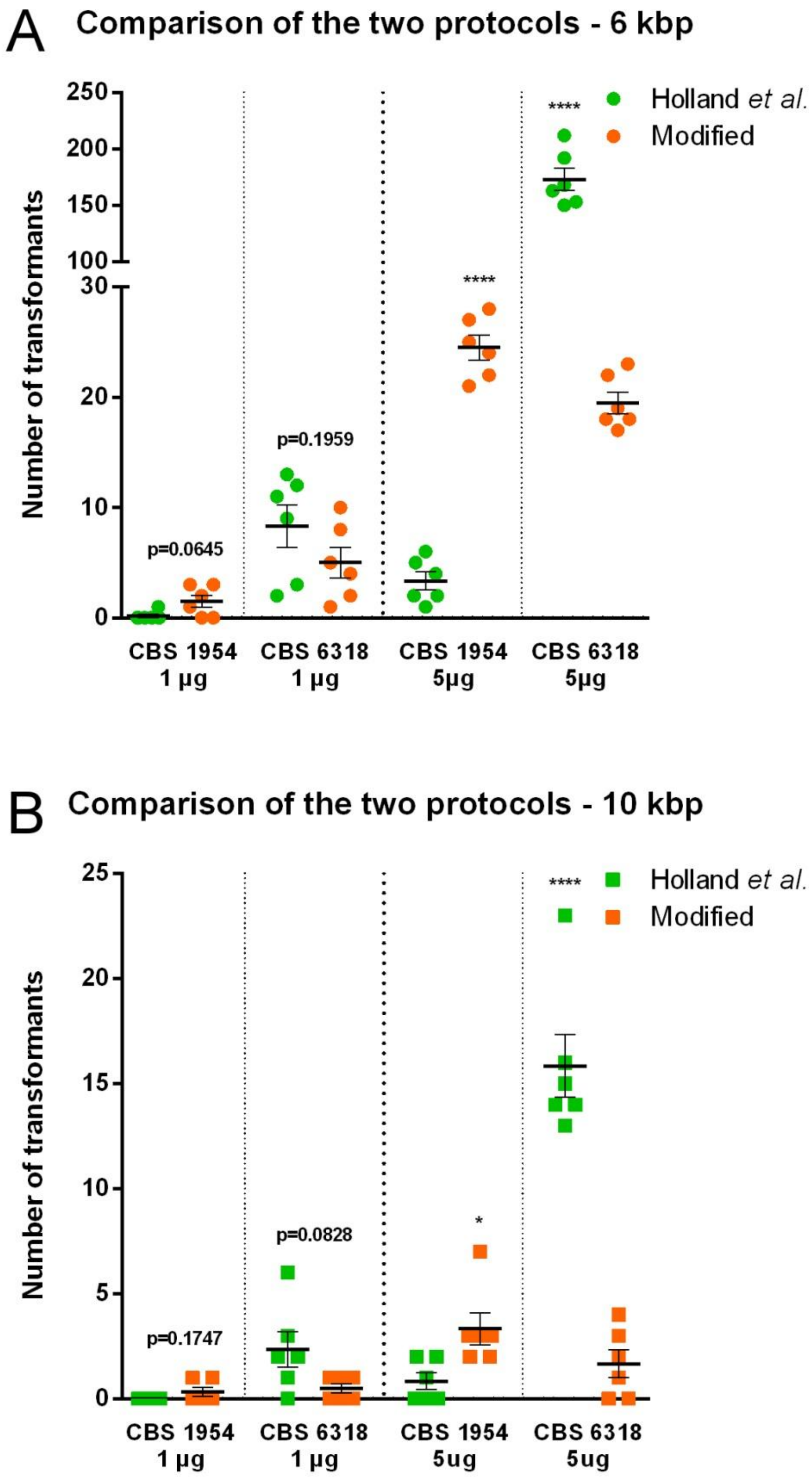

Supplementary Figure S5. Application of our protocol to different *C. parapsilosis* isolates in comparison with the one of Holland *et al*. Where significant difference was not found (CBS 1954 and CBS 6318), the amount of the transforming DNA was increased from one to five µg/transformation. 6kbp (5,657 bp) (Panel A) or 10kbp (9,677 bp) (Panel B) fragments were applied carrying the dominant selectable marker *CaSAT1*. All experiments were performed in three biological parallels with two statistical parallels. Statistical significance was determined by unpaired t test with Welch's correction (* $p < 0.05$; **** $p < 0.0001$).
