# Supplementary material for: Enhancing the chemical transformation of Candida parapsilosis
Source: Virulence. 2021 Mar 17;12(1):937–50. doi: 10.1080/21505594.2021.1893008 (PMC7993187; doi:10.1080/21505594.2021.1893008)
Supplement: Supplemental Material [file KVIR_A_1893008_SM1043.zip › Supplementary material 1.pdf]

Chemical transformation of *Candida parapsilosis* according to Holland *et al.* (2014)

Reagents
Glucose (Biolab)
Peptone (Sigma)
Yeast extract (VWR)
Agar (Sigma)
Distilled water for liquid/solid media
Nourseothricin (NTC) (for dominant selectable marker) (Jena Bioscence)
Yeast nitrogen base (YNB) (for auxotrophic selectable marker) (Sigma)
10x Drop out solution (for auxotrophic selectable marker) (recipe in Supplementary Table S1)
EDTA-Na_2*H_2O (Sigma)
Tris(hydroxymethyl)aminomethane (Sigma)
Lithium-acetate (Sigma)
Polyethylene-glycol 3350 (PEG_{3350}) (Sigma)
Milli-Q bidistilled water
Calf thymus DNA (10 mg/ml stock)*
37 % (m/V) HCl (for pH adjustment) (Molar)
10 M NaOH (for pH adjustment) (Molar)
Transforming DNA

Equipment
1.5 ml microcentrifuge tubes
50 ml conical tubes
Centrifuge for 1.5 ml microcentrifuge tubes
Centrifuge for 50 ml conical tubes
Water bath
Petri plates
Incubator (30 °C)
Spectrophotometer with cuvettes
Sterile box
Glass flask for cultivation
Orbital shaker (30 °C, 150 rpm)
Ice
Pipettes and tips

Arrangements

To prepare 10x TE use 0.5 M EDTA (pH=8) and 2 M tris(hydroxymethyl)aminomethane (pH=8) stock solutions. (Replace these solutions on a three month basis.). Prepare 10x TE, 1 M lithium-acetate, and 50 % (m/V) PEG_{3350} freshly on the day of the transformation. Sterilize solutions in an autoclave and use them as stocks to prepare 1x TELioAc (1/10 volume of 1 M lithium-acetate, 1/10 volume of 10x TE and 8/10 volume of water) and PLATE solution (1/10 volume of 1 M lithium-acetate, 1/10 volume of 10x TE and 8/10 volume of 50 % (m/V) PEG_{3350}). Use Milli-Q water for the solutions and washing the cells. Place 10 µl x (number of transformation + 1) salmon sperm in boiling water in a microcentrifuge tube for 10 minutes and then cool it down rapidly on wet ice (ssDNA) then keep it on ice. Prepare YPD liquid media (1 % (m/V) glucose, 1% (m/V) peptone, 0.5 % (m/V)). Perform experiment at room temperature unless otherwise stated.

Prepare 1.) minimal plates: 0.19 % (m/V) yeast nitrogen base, 2 % (m/V) glucose and 2 % (m/V) agar, or 2.)YPD-NTC plates: YPD liquid media + 2 % (m/V) agar and 200 μg/ml nourseothricin for selection.

1.) Inoculate in 5 ml YPD and incubate overnight at 30 °C (~150 rpm)

2.) On the other day adjust OD_{600} to 0.2 in 50 ml of YPD and incubate at 30 °C (~150 rpm)
3.) When OD_{600} 1.0, harvest the cells in a 50 ml conical tube (~2000 x g, 5 minutes, 4 °C)
4.) Discard supernatant and wash the cells with 3ml ice-cold water (~2000 x g, 5 minutes, 4 °C)
5.) Discard supernatant and resuspend the cells in 1 ml ice-cold 1x TELioAc
6.) Centrifuge in a bench top centrifuge ~17,000 x g, 30 seconds
7.) Remove supernatant and resuspend the pellet in 300 μl ice-cold 1x TELioAc
8.) Set up transformation mixture in a 1.5 ml microcentrifuge tube as follows:
    10 μl ssDNA (10 mg/ml)
    30-50 μl transforming fragment (100 ng/μl)**
    100 μl cell suspension in ice-cold 1x TELioAc from Step 7.)
9.) Shake the tubes gently to combine contents
10.) Incubate at 30 °C for 30 minutes (Static)
11.) Add 700 μl freshly prepared PLATE solution and mix gently
12.) Incubate overnight at 30 °C (Static)***

13.) On the other day perform a heat shock in a water bath (44 °C for 15 minutes)
14.) Centrifuge 17,000 x g, 30 seconds
15.) Carefully remove supernatant
16.) Add 1 ml of fresh YPD without disturbing the pellet
17.) Centrifuge at 17,000 x g, for 30 seconds
18.) Remove supernatant and resuspend the pellet in 100 μl of YPD
19.) Incubate at 30°C with shaking (~150 rpm) for 2 hours (auxotrophic selectable marker) or 4 hours (dominant delectable marker)
20.) Centrifuge at 17,000 x g for 20 seconds
21.) Remove supernatant
22.) Resuspend pellet in 100 μl YPD and plate on selective media
23.) Incubate at 30 °C for 3 days (auxotrophic -) or 2 days (dominant selectable marker)

* We applied salmon sperm (Sigma) instead of calf thymus DNA
** We used 10 μl of transforming DNA (100 ng/μl)
*** We incubated the transformation mixture for 15 hours
