# Supplementary material for: Enhancing the chemical transformation of Candida parapsilosis
Source: Virulence. 2021 Mar 17;12(1):937–50. doi: 10.1080/21505594.2021.1893008 (PMC7993187; doi:10.1080/21505594.2021.1893008)
Supplement: Supplemental Material [file KVIR_A_1893008_SM1043.zip › Supplementary material 2.pdf]

Deposition of *Candida parapsilosis* competent cells

The protocol here describes the solutions and procedures required for freezing and thawing *C. parapsilosis* competent cells for chemical transformation. If one aims to only do either of these procedures, the reagents required for the other are obviously unnecessary to prepare. However, it is strongly suggested to perform a control transformation on the day of the competent cell preparation. This control should be always included when competent cells are taken from the freezer to ensure that competence has not altered. Use competent cell within eight weeks. This protocol relies on the one originally developed by Suga and Hatakeyama (2005).

<u>Reagents</u>

Glucose (Biolab)
Peptone (Sigma)
Yeast extract (VWR)
Agar (Sigma)
Distilled water for liquid/solid media
Nourseothricin (NTC) (for dominant selectable marker) (Jena Bioscence)
Yeast nitrogen base (YNB) (for auxotrophic selectable marker) (Sigma)
10x Drop out solution (for auxotrophic selectable marker) (recipe in Supplementary Table S1)
EDTA-Na_2*H_2O (Sigma)
Tris(hydroxymethyl)aminomethane (Sigma)
Lithium-acetate (Sigma)
Polyethylene-glycol 3350 (PEG_{3350}) (Sigma)
Sterile DMSO (Sigma)
Milli-Q bidistilled water
Salmon sperm DNA (10 mg/ml stock) (Sigma)
37 % (m/V) HCl (for pH adjustment) (Molar)
10 M NaOH (for pH adjustment) (Molar)
Transforming DNA
87 % (V/V) glycerol (Sigma)

<u>Equipment</u>

1.5 ml microcentrifuge tubes
50 ml conical tubes
Centrifuges for 1.5 ml microcentrifuge and 50 ml conical tubes
Water bath
Petri plates
Incubator (30 °C)
Spectrophotometer with cuvettes
Sterile box
Glass flask for cultivation
Orbital shaker (30 °C, 150 rpm)
Ice
Pipettes and tips
- 80 °C freezer
Textile towel
Aluminium foil
Box for microcentrifuge tubes (plastic or paper)

Arrangements for freezing the cells

Prepare YPD liquid media for cultivation: 1 % (m/V) glucose, 1% (m/V) peptone, 0.5 % (m/V) yeast extract and autoclave. To prepare 10x TE use 0.5 M EDTA (pH=7.5) and 2 M tris(hydroxymethyl)aminomethane (pH=8) stocks solutions (final concentrations 10 mM and 100 mM respectively). (Replace the stock solutions on a three month basis.)

Prepare 10x TE and 1 M lithium-acetate and 55 % (m/V) PEG_{3350} freshly on the day of the transformation.

Sterilize these solutions along with 87 % (V/V) glycerol in an autoclave and use as stocks to prepare 1x TELioAc (1/10 volume of 1 M lithium-acetate, 1/10 volume of 10x TE and 8/10 volume of water) 30 % (V/V) glycerol-1x TELioAc is prepared as mixing the followings: 3.45 ml 87 % (V/V) glycerol, 1 ml 10x TE, 1 ml 1 M lithium-acetate and 4.55 ml water. (Scale up or down accordingly).

Use sterile Milli-Q water for the solutions and washing the cells.

Perform experiment at room temperature unless otherwise stated. For *C. parapsilosis* cultivation one can scale up the volume, but the volume of the suspension should not exceed 1/3 of the total volume of the flask.

Arrangements for thawing and transformation

Prepare YPD liquid media for washing the cell pellet and regeneration. Prepare the corresponding plates for selection. For auxotrophic marker: 0.19 g m/V YNB, 2 % m/V glucose, 2 % (m/V) agar, autoclave, and add 10% V/V 10x Drop out solution. For dominant selectable marker: prepare YPD broth supplemented with 2 % m/V agar, autoclave and add nourseothricin in a final concentration of 200 µg/ml.

Use freshly prepared and autoclaved 10x TE and 1 M lithium-acetate (see above) to prepare PLATE(3350) solution (1/10 volume of 1 M lithium-acetate, 1/10 volume of 10x TE, 8/10 volume of 55 % (m/V) PEG_{3350}. Mix this solution with sterile DMSO in a ratio of 10:1 and mix vigorously to obtain +DMSO/PLATE(3350). Protect the mixture from light by covering the tube containing the solution with aluminium foil.

Place 10 µl x (number of transformation + 1) salmon sperm in boiling water in a microcentrifuge tube for 10 minutes and then cool it down rapidly on ice (ssDNA) then keep it on ice. (+1 is needed to ensure that salmon sperm DNA will be enough.)

Perform experiment at room temperature unless otherwise stated.

Procedure

1.) Inoculate the strain in 5 ml YPD and incubate overnight at 30 °C (~150 rpm)

2.) On the other day adjust OD_{600} to 0.05 in 150 ml of YPD (in a 500 ml flask) and incubate at 30 °C (~150 rpm)

3.) When OD_{600} = 1.34 ± 0.05, transfer the suspension in 50 ml conical tubes

4.) Centrifuge at ~2000 g, 5 minutes

5.) Discard supernatants and wash the cells with (25-25 ml) of water (~2000 x g, 5 minutes)

6.) Discard supernatants and suspend the cells in a total of 5 ml of 1x TELioAc and transfer the suspensions in a single 50 ml conical tube

8.) Centrifuge at ~2000 x g, 5 minutes

9.) Remove supernatant and suspend the pellet in 3.7 ml 1x TELioAc containing 30 % (V/V) glycerol

10.) Aliquot 50-50 µl of this suspension to sterile microcentrifuge tubes (~75 pieces), mix the content of the conical tube often to keep the suspension homogenous

11.) Place the tubes in a microcentrifuge box (paper or plastic), cover it with two layers of textile towel and place it into a -80 °C freezer

Thawing and transformation

1.) Set up the transformation mixtures in 1.5 ml microcentrifuge tubes on ice as follows (include a control):

10 µl ssDNA (10 mg/ml)

10 µl transforming fragment

Mix contents, then spin the tubes down in a bench top centrifuge and keep them on ice

2.) Turn on a water bath and set it to 30 °C

3.) Take as much vials **+1** you need from the -80 °C freezer (**+1** is for control)

4.) Place the tubes in a floater and then into the water bath (30 °C) for 15 seconds

5.) Settle the cells by flicking the tube rapidly, but gently downward

6.) Place the tubes back into the water bath for another 15 seconds

7.) Spin down the suspension gently in a bench top centrifuge

8.) Carefully suspend the pellet and add to the transformation mixture from Step 1.)

9.) Gently flick the tubes to mix contents

10.) Add 770 µl freshly prepared DMSO/PLATE(3350) solution immediately, then turn the tubes upside down and flick them gently three times to mix contents

11.) Incubate for 15 hours at 30 °C overnight (Static)

12.) On the other day perform a heat shock in a water bath (44 °C for 15 minutes)

13.) Centrifuge 17,000 x g, 1 minute

14.) Carefully remove as much supernatant as possible

15.) Add 950 µl of fresh YPD without disturbing the pellet, then turn the tubes upside down and flick them gently once

16.) Centrifuge at 17,000 x g, for 1 minute

17.) Remove as much supernatant as possible and suspend the pellet in 300 µl of YPD

18.) Incubate at 30°C with shaking (~ 150 rpm) for 2 hours (auxotrophic selectable marker) or 4 hours (dominant selectable marker)

19.) Centrifuge at 2,400 x g for 3 minutes

20.) Remove 150 µl of supernatant

21.) Suspend the pellet in the rest of the supernatant and plate onto selective media

22.) Incubate at 30 °C for 3 days (auxotrophic -) or 2 days (dominant selectable marker)
